# Effective Cellular Morphology Analysis for Differentiation Processes by a Fluorescent 1,3a,6a-Triazapentalene Derivative Probe in Live Cells

**DOI:** 10.1371/journal.pone.0160625

**Published:** 2016-08-04

**Authors:** Rui Kamada, Fumi Tano, Fuki Kudoh, Nozomi Kimura, Yoshiro Chuman, Ayumi Osawa, Kosuke Namba, Keiji Tanino, Kazuyasu Sakaguchi

**Affiliations:** 1 Laboratory of Biological Chemistry, Department of Chemistry, Faculty of Science, Hokkaido University, Sapporo, Japan; 2 Laboratory of Organic Chemistry II, Department of Chemistry, Faculty of Science, Hokkaido University, Sapporo, Japan; 3 Graduate School of Pharmaceutical Science, The University of Tokushima, Tokushima, Japan; University of Toronto, CANADA

## Abstract

Nuclear and cytoplasmic morphological changes provide important information about cell differentiation processes, cell functions, and signal responses. There is a strong desire to develop a rapid and simple method for visualizing cytoplasmic and nuclear morphology. Here, we developed a novel and rapid method for probing cellular morphological changes of live cell differentiation process by a fluorescent probe, TAP-4PH, a 1,3a,6a-triazapentalene derivative. TAP-4PH showed high fluorescence in cytoplasmic area, and visualized cytoplasmic and nuclear morphological changes of live cells during differentiation. We demonstrated that TAP-4PH visualized dendritic axon and spine formation in neuronal differentiation, and nuclear structural changes during neutrophilic differentiation. We also showed that the utility of TAP-4PH for visualization of cytoplasmic and nuclear morphologies of various type of live cells. Our visualizing method has no toxicity and no influence on the cellular differentiation and function. The cell morphology can be rapidly observed after addition of TAP-4PH and can continue to be observed in the presence of TAP-4PH in cell culture medium. Moreover, TAP-4PH can be easily removed after observation by washing for subsequent biological assay. Taken together, these results demonstrate that our visualization method is a powerful tool to probe differentiation processes before subsequent biological assay in live cells.

## Introduction

Cells regulate nuclear and cellular structures, such as shape and size, in response to signals and differentiation. All tissues constituting organs differentiate from stem cells. Deficient or abnormal differentiation frequently causes severe diseases. Morphological changes of the nucleus have been observed in most cancers. Alterations of nuclear morphology, including the size and shape, are characteristic of the tumor type and stage [1]. Thus, analyzing nuclear morphological changes is important for cancer diagnosis. In the field of hematology, analyzing the shape and size of the nucleus and cytoplasm is an essential step to distinguish various types of cells [2]. The morphological changes of leukocytes, such as neutrophils and monocytes, can provide important information about the differentiation and pathologies of diseases such as leukemia [3]. In addition, analyzing neuronal morphology, including axons and dendrites, is important to understand the functions and differentiation of neurons and is required for diagnosis [4]. Therefore, analyzing cytoplasmic and nuclear morphologies in live cells is required for cancer diagnosis and understanding cellular functions, signal responses, and differentiation processes.

To observe cellular morphological changes, specific visualization probes are required for the cytoplasm and/or nucleus. There are many chemical fluorescent probes that specifically stain/visualize cellular organelles such as the cell membrane, nucleus, Golgi apparatus, endoplasmic reticulum (ER)[5], mitochondria, and lysosomes [6]. However, there are few reports of cytoplasmic specific visualization probes [7–9]. Moreover, there are no suitable chemical probes that simultaneously visualize both cytoplasmic and nuclear morphological changes before subsequent biological analysis. A compound targeting the cytoskeleton can visualize cellular morphology, but it is unable to provide information about nuclear morphology [10]. One of the advantages of a specific visualization probe for the cytoplasm is observation of both the cytoplasm and nucleus. To date, fusion fluorescent proteins and immunofluorescence labeling with an antibody against proteins localized in the cytoplasm are used to visualize cytoplasm [11, 12]. However, it is not easy to introduce an expression plasmid into certain cell types including non-adherent cells, primary cells, and stem cells. Furthermore, DNA transfection itself influences the cells. Thus, there is a strong demand for development of an efficient small chemical probe to visualize cytoplasmic and nuclear morphologies in living cells.

Most of the previously reported chemical probes stain the cell organelles by irreversible processes. The cells, which are stained by these probes, are difficult to be applied for other biological analysis. Because biological samples collected from patients or animal models are quite limited in cell number, transient and harmless visualization method is required. Here, we report a novel fluorescent probe, a 1,3a,6a-triazapentalene derivative with a 4-biphenyl group, namely TAP-4PH, as a powerful tool to transiently observe cytoplasmic and nuclear morphological changes in various types of live cells. Our novel and rapid method to visualize cellular morphological changes by TAP-4PH enable us to probe cellular differentiation processes in live cells before subsequent biological assay.

## Materials and Methods

### 1.1. Cell culture

A549 human lung adenocarcinoma cells, H1299 human non-small cell lung carcinoma cells, HeLa human cervical cancer cells, NT2 human teratocarcinoma cells, and HEK293 human embryonic kidney cells were obtained from ATCC (Rockville, MD, USA) and grown in Dulbecco’s modified Eagles medium (DMEM) supplemented with 10% FBS and penicillin/streptomycin in a humidified atmosphere with 5% CO_2_. HL-60 human promyelocytic leukemia cells and PC-12 rat pheochromocytoma cells were obtained from ATCC (Rockville, MD, USA) and grown in RPMI-1640 medium supplemented with 10% FBS and penicillin/streptomycin or RPMI-1640 medium supplemented with 10% horse serum, 5% FBS, and penicillin/streptomycin. 3T3-L1 mouse fibroblasts were obtained from the Japanese Collection of Research Bioresource Bank (Tokyo, Japan) and grown in DMEM supplemented with 10% bovine calf serum and penicillin/streptomycin.

### 1.2. Cell differentiation (PC-12, HL-60, and 3T3-L1)

PC-12 cells (1 × 10^5^ cells) were seeded onto a type-IV collagen (Nitta Gelatin, Osaka, Japan)-coated 35-mm glass bottom dish (Iwaki Glass, Chiba, Japan) with 2 ml of medium and incubated for 24 h prior to differentiation. The cells were then induced to undergo neuronal differentiation in RPMI-1640 medium containing 0.1% horse serum, 0.05% FBS, penicillin/streptomycin, and 50 nM NGF (BD Biosciences).

HL-60 cells (1 × 10^6^ cells) were seeded onto a 100-mm tissue culture dish with 10 ml of medium or a μ-Dish 35-mm, ibiTreat, tissue culture-treated dish (ibidi, Munich, Germany) with 2 ml of medium and incubated for 24 h before differentiation. The cells were then treated with 1 μM all-trans retinoic acid (ATRA) (Sigma-Aldrich, St. Louis, MO) or 16 nM phorbol 12-myristate 13-acetate (PMA) (Sigma-Aldrich) to induce differentiation into neutrophil- or macrophage-like cells for the indicated time.

3T3-L1 cells (1 × 10^5^ cells) were seeded onto a μ-Dish 35-mm, ibiTreat, tissue culture-treated dish and cultured to confluency. After 48 h of culture, the cells were induced to differentiate into adipocytes by incubation in DMEM supplemented with 10% FBS, 1 μM dexamethasone (Wako Chemical Industries, Ltd., Osaka, Japan), 0.5 mM methylisobutylxanthine (Sigma), and 1 μg/ml insulin (Sigma-Aldrich).

### 1.3. Fluorescence spectroscopy

Fluorescence spectra of TAP-4PH (10 μM) in the indicated solutions were measured using a Fluorescence Spectrophotometer F-4500 (Hitachi, Ltd., Tokyo, Japan) under the following conditions. Excitation wavelength: 318 nm; scan speed: 1200 nm/min; excitation and emission slit: 5 nm; PMT voltage: 700 V. If necessary, salmon sperm DNA was added.

### 1.4. Fluorescence Microscopy analysis

Cells were seeded onto a μ-Dish 35-mm, ibiTreat, tissue culture-treated dish and cultured for 24 h prior to treatment. TAP-4PH was added to the culture medium, followed by incubation for the indicated times. The cells were then observed by fluorescence microscopy. Cellular organelle co-staining with the nucleus by acridine orange (Life Technologies, Carlsbad, CA) or Nuclear Green LCS1 (abcam, Cambridge, MA), the Golgi apparatus by ViVidFluor Golgi Green/Red (Wako Chemical Industries, Ltd., Osaka, Japan), and ER by ER Tracker Red (Thermo Fisher Scientific, NH) was performed in accordance with the manufacturers’ instructions before addition of TAP-4PH to the medium. After staining with the indicated organelle probes, the cells were treated with TAP-4PH for 30 min and then observed under a fluorescence microscope (Biorevo BZ-9000, Keyence, Osaka, Japan) using the following filter set: DAPI-B (Ex360/40 Dm400 Em460/50) for detection of TAP-4PH, GFP (Ex470/40 Dm495 Em535/50) for detection of Golgi Green/Red, and TRITIC (Ex540/25 Dm565 Em605/55) for detection of acridine orange and ER tracker red.

### 1.5. Cell proliferation assay

Cells (1 × 10^4^/well) were seeded onto 96-well flat-bottomed plates with 100 μl of medium and incubated for 24 h before TAP-4PH treatment. TAP-4PH was added at 10–50 μM, followed by 24 h of incubation. Cell viability was then measured by a Burker-truk line (Erma, Japan).

### 1.6. Flow cytometric analysis

HL-60 cells before and after differentiation into neutrophil-like cells were collected and washed with PBS containing 2% FBS. The cells were treated with 50 μM TAP-4PH for 30 min at 37°C and then analyzed by flow cytometry (Gallios; Beckman Coulter, Miami, FL). To quantify cellular uptake of TAP-4PH, 1 × 10^6^ HL-60 cells were seeded onto a 35-mm tissue culture dish with 2 ml of medium and incubated for 24 h. The cells were washed with PBS containing 2% FBS and then incubated at 4°C for 30 min. TAP-4PH was added at a final concentration of 50 μM, followed by incubation for 30 min at 4°C. The cells were then collected and analyzed by the Gallios flow cytometer.

### 1.7. Phagocytosis assay

The phagocytosis assay of HL-60 cells was performed using a Phagocytosis Assay kit (Cayman Chemical CO., Ann Arbor, MI) according to the manufacturer’s protocol. Briefly, HL-60 cells were differentiated into neutrophil-like cells with 1 μM ATRA for 5 days. The cells were treated with 50 μM TAP-4PH for 30 min, followed by washing to remove TAP-4PH from the cells. Cells (1 × 10^6^/well) were seeded onto 24-well plates with 2 ml of medium containing Latex IgG-PE beads. After 24 h of incubation, the cells were collected and analyzed by the Gallios flow cytometer.

### 1.8. Cell cycle analysis

Cell cycle analysis was performed as described previously [13]. Briefly, 1 × 10^6^ HL-60 cells were seeded on a 10-cm tissue culture dish with 10 ml of medium and incubated for 24 h before TAP-4PH treatment. TAP-4PH was added to the culture medium at 10 μM, followed by incubation for 48 h. The cells were then fixed and stained with PI/RNase buffer (BD Pharmingen, San Diego, CA). DNA cell cycle analysis was performed by the Gallios flow cytometer.

## Results

**Fig 1:**
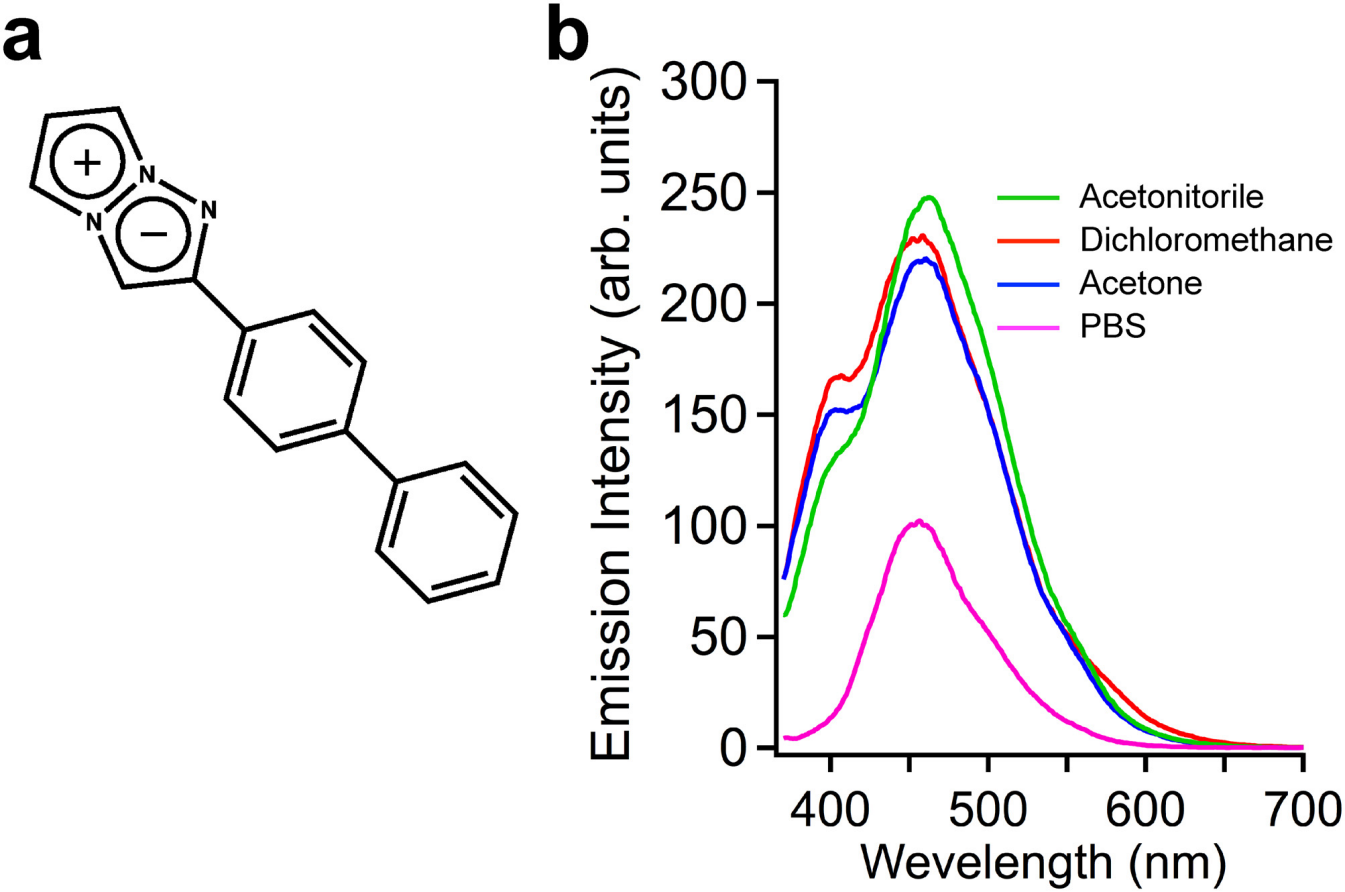
Chemical structure and fluorescence emission spectra of TAP-4PH. a) Chemical structure of the 1,3a,6a-Triazapentalene derivative TAP-4PH. b) Fluorescence emission spectra of TAP-4PH (10 μM) in aqueous and organic solvents. Green: acetonitrile; red: dichloromethane; blue: acetone; magenta: PBS.

To apply a fluorescent probe to cell biological applications, the fluorescent compound should be water soluble and small for cellular uptake and emit high fluorescence in an aqueous solvent. We have previously designed and synthesized fluorescent 1,3a,6a-triazapentalene derivatives (Fig 1a) that have a wide range of emission wavelengths [14–16]. Unlike other fluorescent probes with a relatively large molecular size, 1,3a,6a-triazapentalenes have a unique compact skeleton with a 10π-electron system and highly fluorescent chromophore. By applying 2- and 5-substituents, 1,3a,6a-triazapentalenes can be tuned for both fluorescence wavelength and quantum yield. In addition, 1,3a,6a-triazapentalene can be synthesized by our efficient method with an intermolecular cyclo-addition reaction in a single step.

**Fig 2:**
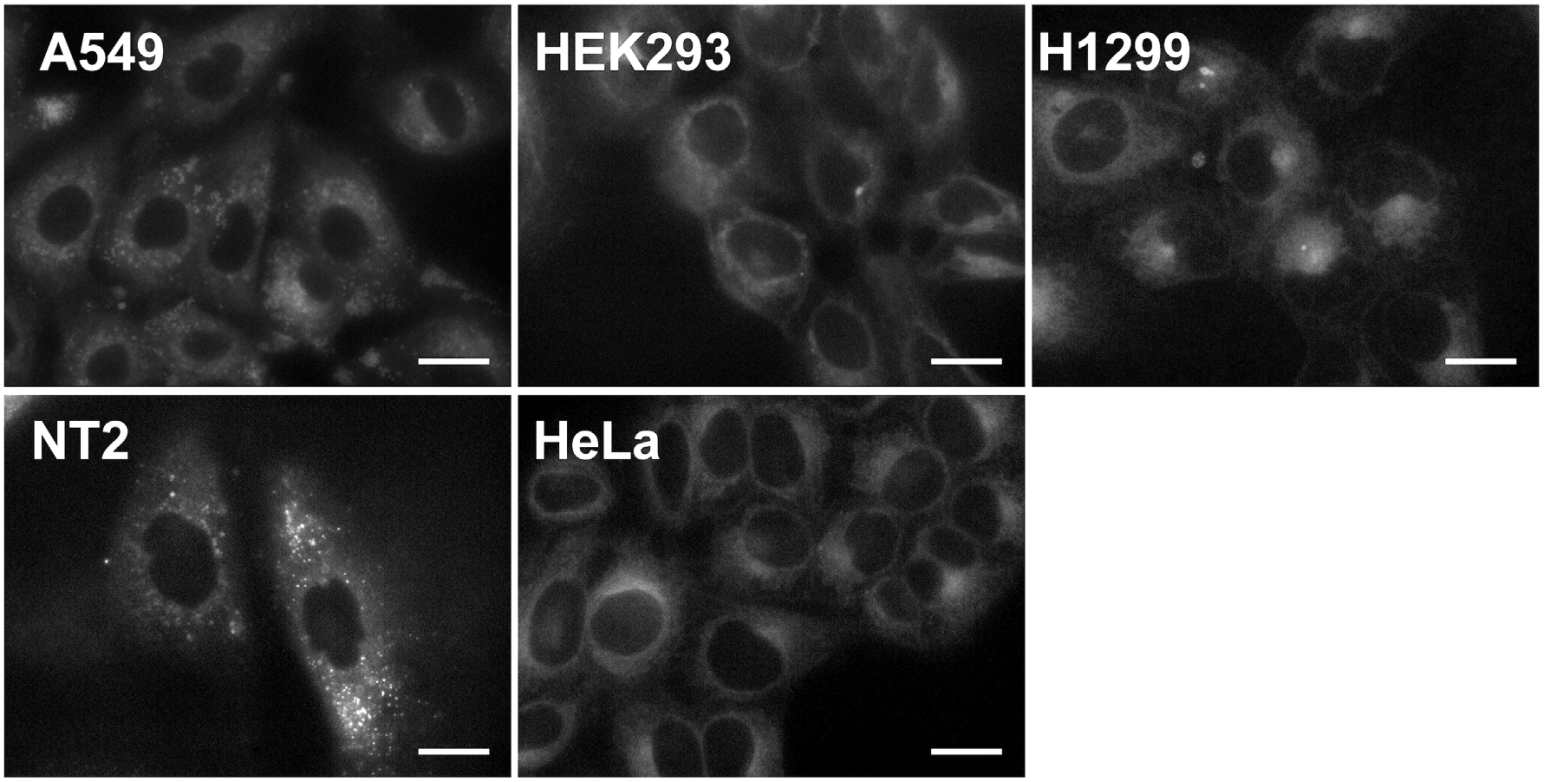
Live cell visualization of cytoplasm by TAP-4PH in various cell types. Luminescence images of live cells treated with 10 μM TAP-4PH for 30 min at 37°C. Scale bar: 20 μm.

**Fig 3:**
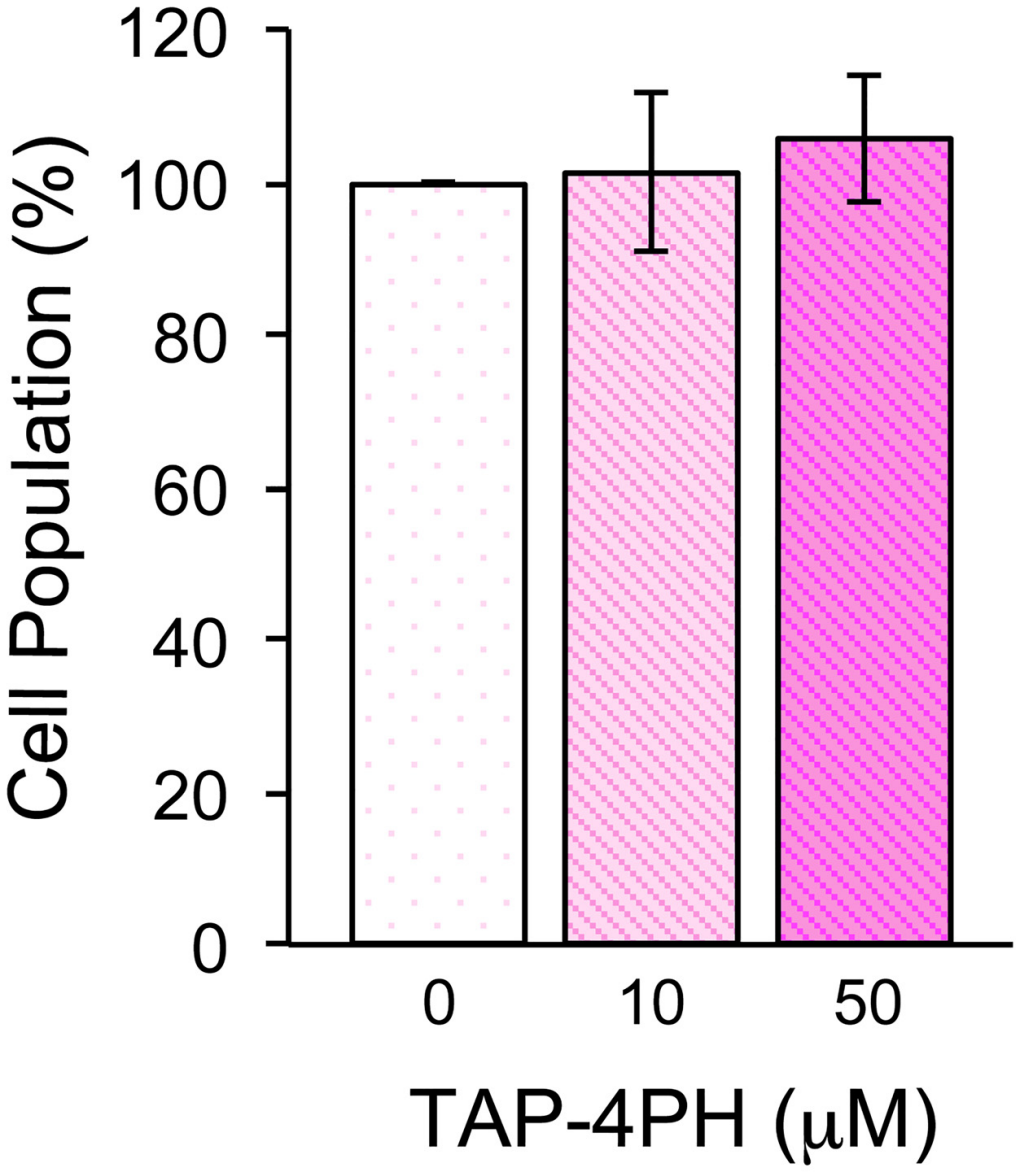
Cell proliferation after treatment of A549 cells with TAP-4PH. Cell numbers were measured after treatment with TAP-4PH for 24 h. Data represent the mean ± SE, n = 3.

The fluorescence excitation spectra of TAP-4PH were measured in various organic solvents and water (Fig 1b). TAP-4PH showed high fluorescence in phosphate-buffered saline (PBS) and organic solvents (λ_ex_/λ_em_ = 345 nm/456 nm). The fluorescence intensity in PBS was about half of that in dichloromethane (DCM). Next, various types of cells, tumor-derived cell lines (human lung cancer-derived A549, human cervical cancer-derived HeLa, human non-small cell lung carcinoma-derived H1299, human germ cell embryonal carcinoma-derived NT2/D1) and the human embryonic kidney cell line HEK293, were incubated with 10 μM TAP-4PH for 30 min at 37°C in fetal bovine serum (FBS)-containing medium and then observed by fluorescence microscopy (Biorevo BZ-9000, Keyence, Osaka, Japan). We found that TAP-4PH selectively visualized the cytoplasm of all tested live cells (Fig 2). Co-staining with a nuclear-specific probe confirmed that TAP-4PH selectively visualized the cytoplasm (S1 Fig). The cytotoxicity of TAP-4PH in cells was analyzed by cell counting (Fig 3). TAP-4PH at 10 and 50 μM showed no effect on the proliferation of A549 cells, indicating that TAP-4PH was suitable for live cell imaging.

**Fig 4:**
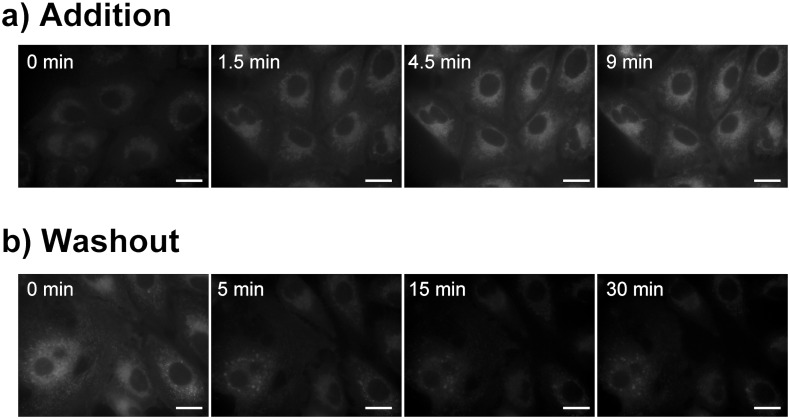
Time-dependent incorporation and distribution of TAP-4PH in A549 cells. a) Luminescence images of A549 cells incubated with 10 μM TAP-4PH for the indicated time periods. b) After incubation with 10 μM TAP-4PH for 30 min, the cells were washed and then incubated in fresh medium without TAP-4PH (time 0 min). Confocal luminescence images were taken after incubation for the indicated time periods. Scale bar: 20 μm.

To analyze time-dependent incorporation of TAP-4PH, A549 cells were incubated with 10 μM TAP-4PH in culture medium for various times. As shown in Fig 4a, the fluorescence of TAP-4PH was detected within 4.5 min. This result indicated that TAP-4PH was incorporated into cells, and the fluorescence was detected in the cytoplasm within 5 min of incubation. The TAP-4PH-treated A549 cells were washed with PBS three times and cultured in fresh medium without TAP-4PH. After 5, 15, and 30 min, we obtained fluorescence images (Fig 4b). The fluorescence intensity was decreased at 30 min after removing TAP-4PH from the culture medium. This result indicated that the TAP-4PH incorporated into cells was removed by simply removing the compound from the medium.

**Fig 5:**
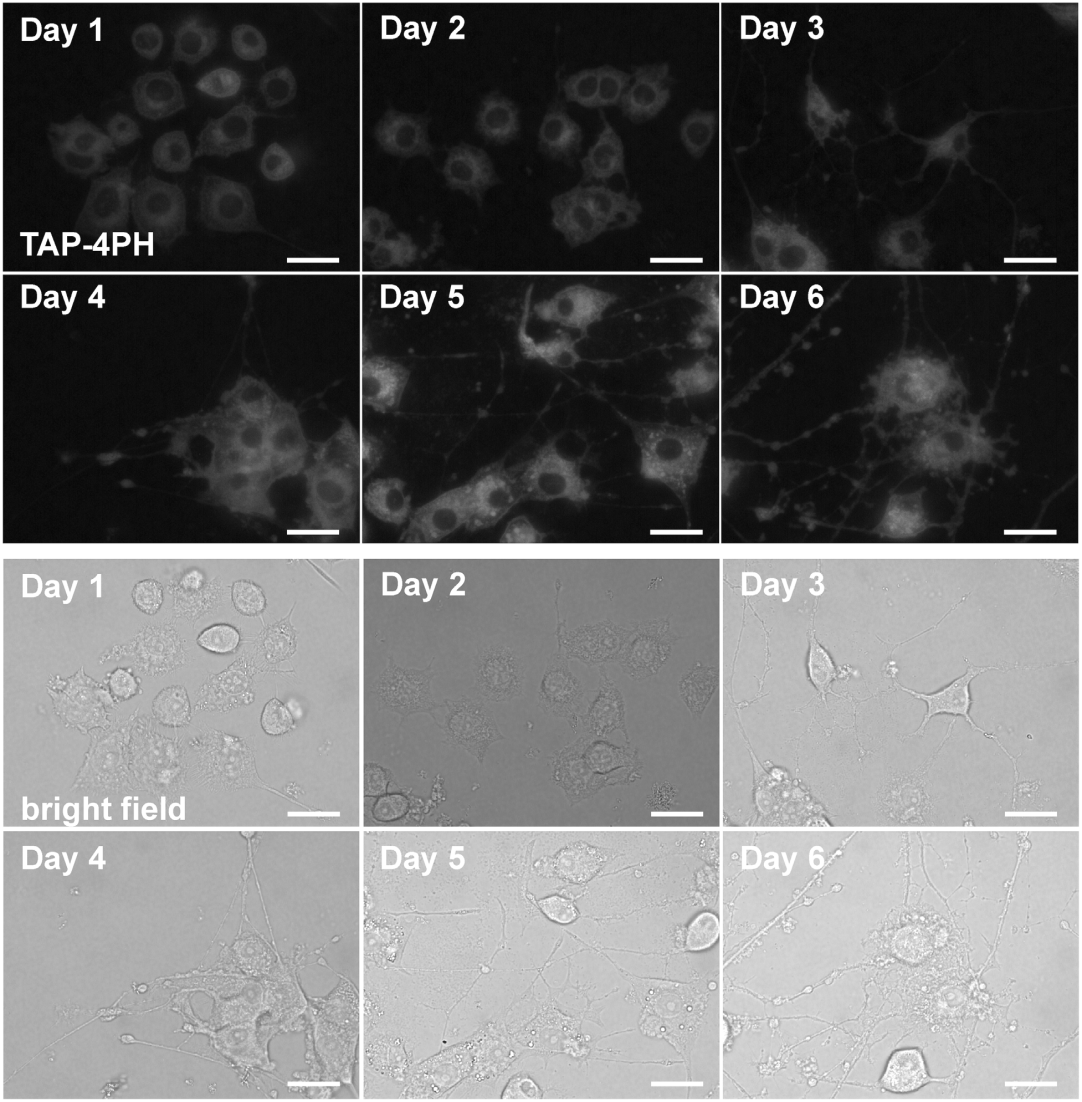
Fluorescence imaging of live cells during neuronal differentiation of PC-12 cells. PC-12 cells were induced to differentiate into nerve cells by NGF. The cells at the indicated days after differentiation were treated with 50 μM TAP-4PH for 30 min. Top panels, TAP-4PH; bottom panels, bright-field images. Scale bar: 20 μm.

To apply TAP-4PH imaging to analyze cell differentiation, we selected the rat pheochromocytoma cell line PC-12 that can be differentiated into sympathetic neuron-like cells [17]. It is important to visualize synapse formation and axonal regeneration to analyze the functions of neuronal cells. However, small chemical probes, which stain and visualize synapses and axons in live cells without toxicity, are not available. Therefore, immunostaining or transfection of a fluorescent protein needs to be employed for visualization. PC-12 cells were induced to differentiate by nerve growth factor (NGF). The cells at the indicated days after incubation with NGF were treated with 50 μM TAP-4PH and then observed by fluorescence microscopy. As shown in Fig 5 and S2 Fig, TAP-4PH fluorescence was detected from the cytoplasm to the axons and synapses of differentiated PC-12 cells. Moreover, our probe visualized dendritic spines. It has been reported that dendritic spines act as chemically and electrically segregated microcompartments [18, 19]. Thus, observing dendritic spine formation and morphology is important to analyze synapse functions including long-term plasticity. This is the first demonstration of visualizing axons and synapses, including dendritic spines, by simply adding a small fluorescent compound.

**Fig 6:**
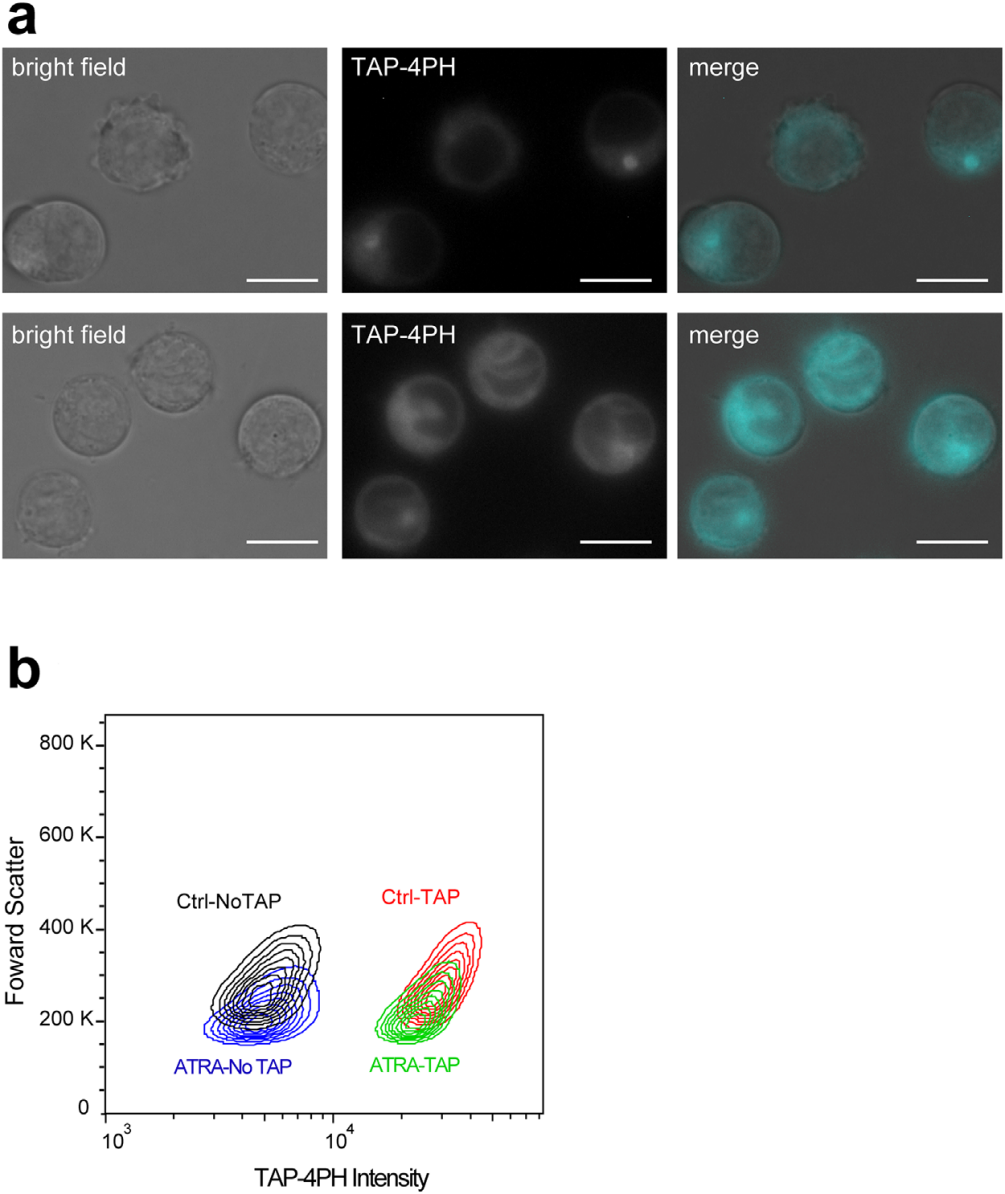
Fluorescence imaging and flow cytometric analysis of live cells during neutrophilic differentiation of HL-60 cells. a) HL-60 cells were differentiated into neutrophil-like cells by ATRA for 6 days. The cells were treated with 50 μM TAP-4PH for 30 min. Left panels, bright field; middle panels, TAP-4PH; right panels, merged images of bright field and TAP-4PH (cyan). Scale bar: 10 μm. b) Density plot showing TAP-4PH fluorescence and forward scatter in HL-60 cells before (left) and after (right) ATRA-induced differentiation. Black: undifferentiated HL-60 cells without TAP-4PH; blue: differentiated HL-60 cells without TAP-4PH; red: undifferentiated HL-60 cells treated with 50 μM TAP-4PH; green: differentiated HL-60 cells treated with 50 μM TAP-4PH.

We applied our TAP-4PH visualization method to observe both cytoplasmic and nuclear morphological changes in response to differentiation of the human promyelocytic leukemia cell line HL-60. Because TAP-4PH showed high fluorescence at cytoplasmic area, TAP-4PH can visualize both cytoplasmic and nuclear morphology at the same time. HL-60 cells can be induced to differentiate into neutrophil- and macrophage-like cells by ATRA and PMA, respectively [20–23]. The HL-60 cells were treated with 1 μM ATRA for 5 days to differentiate into neutrophil-like cells, and then their cytoplasm was visualized with 50 μM TAP-4PH (Fig 6a). ATRA treatment of HL-60 cells is known to cause dramatic morphological changes in the nuclear structure from rounded nuclei to segmented or banded nuclei, which are characteristics of neutrophil myelocytes. The TAP-4PH visualization method clearly determined nuclear structural changes from the rounded nuclei of undifferentiated cells to the segmented or banded nuclei of neutrophil-like cells (S3a Fig). In addition, unlike simple nuclear staining, TAP-4PH also visualized the structure and size of the cytoplasm. We also detected the change in cell size from undifferentiated cells to differentiated neutrophil-like cells. In the case of macrophage-like differentiated HL-60 cells, TAP-4PH also visualized the cytoplasm of attached macrophage-like HL-60 cells (S3b Fig). Our TAP-4PH staining method is a very simple and efficient detection method for differentiation of cells.

Flow cytometric analysis of TAP-4PH-treated HL-60 cells was carried out. TAP-4PH fluorescence was found in each undifferentiated HL-60 cell compared with control cells without TAP-4PH (Fig 6b). Interestingly, ATRA-induced differentiated HL-60 cells showed weaker intracellular luminescence than undifferentiated HL-60 cells. In response to differentiation into neutrophil-like cells, there was a decrease in the size of HL-60 cells. Therefore, TAP-4PH fluorescence from differentiated neutrophil-like cells might be lower than that from undifferentiated cells.

**Fig 7:**
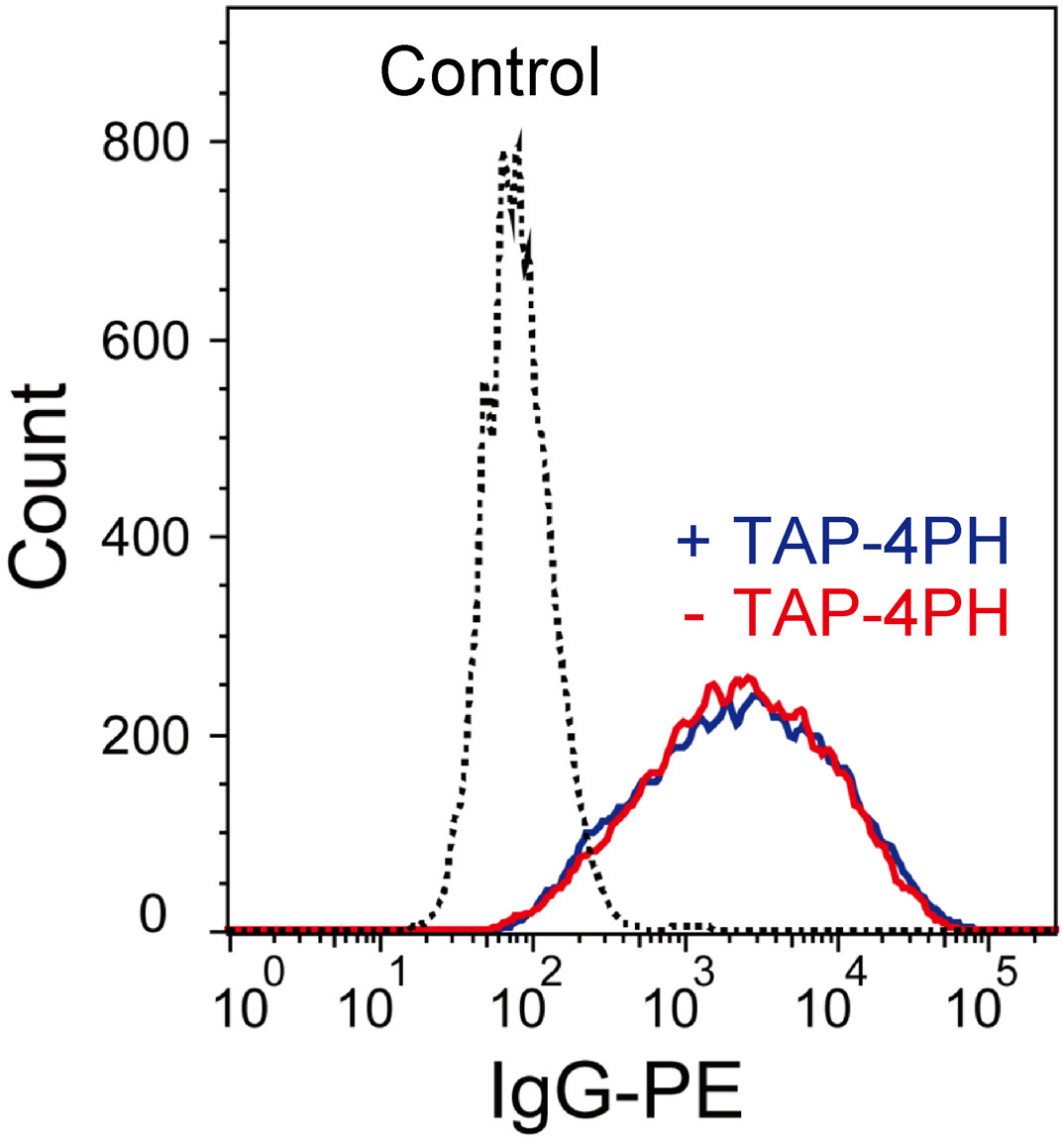
Phagocytosis assay using IgG-coated PE beads. Representative histograms of PE fluorescence in differentiated HL-60 cells without (red) and with (blue) TAP-4PH treatment. HL-60 cells in the absence of IgG-coated PE beads are shown in the black-dotted line.

After observing differentiation of the cells by TAP-4PH treatment, the compound was removed from the cells that continued to grow without cytotoxicity (S4 Fig). To analyze the effect of TAP-4PH visualization on the biological functions of cells, we compared the phagocytosis activity of differentiated HL-60 cells with and without TAP-4PH treatment. After differentiation of HL-60 cells into neutrophil-like cells using ATRA, the cells were incubated with 50 μM TAP-4PH for 30 min. The cells were washed with PBS three times and then incubated in fresh medium with IgG-PE beads. After 24 h of incubation, the cells were analyzed by flow cytometry (Fig 7). The phagocytosis activity of HL-60 cells pre-treated with TAP-4PH was same as that of HL-60 cells without TAP-4PH. This result indicated that TAP-4PH has no effect on cell viability or cellular functions. These properties of TAP-4PH are very useful for transient cellular visualization. After addition of TAP-4PH and observing the cells, the probe can be easily removed from the cells that continue to culture for subsequent biological analysis. Taken together, our rapid and simple visualization method of cytoplasmic and nuclear morphologies by TAP-4PH is a useful method to evaluate cell differentiation processes.

## Discussion

There have been some reports of cytoplasmic fluorescent probes [7–9]. However, the visualization efficiency of these probes is not high and cannot clearly visualize the edge of cells. Therefore, it is probably difficult to observe detailed morphological changes, especially the axons and dendritic spines of neurons. Recently, a specific fluorescent probe to visualize neuronal cells was developed by Er *et al*. [24]. Our probe can visualize both cytoplasmic and nuclear morphological changes in live cells at the same time. We successfully visualized the formation of axons and dendritic spines of PC-12 cells during neuronal differentiation, as well as nuclear segmentation of live HL-60 cells during neutrophilic differentiation using TAP-4PH. Furthermore, after observing the cells, they could be cultured further to perform other biological assays. The cell number can be limited in biological samples, especially in those collected from patients or animal models. Thus, a small number of cells must be analyzed, which are often re-used for other biological analyses after cell visualization/staining.

To understand the mechanism of cytoplasmic and nuclear visualization by TAP-4PH, temperature-dependent cellular uptake of TAP-4PH was analyzed by flow cytometry (S5 Fig). HL-60 cells incubated with TAP-4PH at a low temperature (4°C) exhibited lower cellular uptake of TAP-4PH compared with incubation at 37°C. These results suggested that TAP-4PH entered the cytoplasm in an energy-dependent manner. To further analyze the mechanism of TAP-4PH visualization of cellular morphology, we performed co-staining with a commercial Golgi marker and ER marker. TAP-4PH showed higher fluorescence in the Golgi and ER. However, the fluorescence pattern did not completely match with those of the Golgi and ER probes (S6 Fig). TAP-4PH showed a ~2-fold higher fluorescence intensity in an organic solvent than in an aqueous solvent (Fig 1). After entering cells, TAP-4PH showed higher fluorescence in the cytoplasm, including the Golgi and ER, than in the culture medium, probably because the hydrophobicity of the cytoplasm is higher than that of the medium. TAP-4PH exhibited strong fluorescence in lipid droplets of differentiated 3T3-L1 adipocytes (S7 Fig). Lipid droplets are intracellular energy storage organelles consisting of a hydrophobic core of triglycerides and steryl esters. These results also support that TAP-4PH can visualize cellular morphology because of its fluorescence properties, namely exhibiting higher fluorescence in a hydrophobic environment than in a hydrophilic environment. TAP-4PH fluorescence was not observed in the nucleus, suggesting that TAP-4PH cannot enter the nucleus or the hydrophobicity of the nucleus is lower than that of the cytoplasm. We noted that the fluorescence emission spectrum of TAP-4PH in the presence of DNA showed no difference from that without DNA, suggesting that TAP-4PH was not quenched in the nucleus (S8 Fig). However, further studies will be required to understand the [mechanism of cytoplasmic and nuclear visualization by TAP-4PH.

In conclusion, we have demonstrated that our novel and rapid cellular visualization method by a 1,3a,6a-triazapentalene derivative, TAP-4PH, can monitor cell differentiation processes without toxicity before subsequent biological assay. The utility of TAP-4PH for visualization of cytoplasmic and nuclear morphologies of live cells was demonstrated in various cell types. In addition, TAP-4PH can be removed after observation without any influence on cellular function. Taken together, TAP-4PH provides an easy and powerful tool to observe and monitor live cell differentiation processes.

## Supporting Information

S1 Fig Co-staining with the nuclear staining probe acridine orange and TAP-4PH in A549 cells. Left panel, TAP-4PH; middle panel, acridine orange; right panel, merged images of TAP-4PH (cyan) and acridine orange (magenta). Scale bar: 20 μm.
(TIF)

S2 Fig TAP-4PH visualization of PC-12 cells. Merged bright-field and TAP-4PH fluorescent (cyan) images of PC-12 cells.
(TIF)

S3 Fig TAP-4PH visualization of HL-60 cells. (a) HL-60 cells before and after differentiation into neutrophil-like cells by ATRA for 5 days. After differentiation, the cells were co-stained with Nuclear Green LSC1 for nucleus and TAP-4PH. Left panels, TAP-4PH; middle panels, Nuclear Green LSC1; right panels, merged images of TAP-4PH (cyan) and Nuclear Green LSC1 (yellow). (b) HL-60 cells before and after differentiation into macrophage-like cells by PMA for 48 h. After differentiation, the cells were treated with 50 μM TAP-4PH for 30 min and then observed by fluorescence microscopy. Left panel, bright field; middle panel, TAP-4PH; right panel, merged image of bright field and TAP-4PH (cyan). Scale bar: 20 μm.
(TIF)

S4 Fig Cell cycle analysis of HL-60 cells after incubation for 48 h in the presence (left) or absence (right) of 10 or 50 μM TAP-4PH. Data shown are representative of three independent experiments.
(TIF)

S5 Fig Temperature-dependent cellular uptake of TAP-4PH. HL-60 cells were incubated with 50 μM TAP-4PH for 30 min at 37 or 4°C. Cellular uptake of TAP-4PH was measured by flow cytometric analysis. Data represent the mean ± S.D., n = 3. **p < 0.005, Student’s t-test.
(TIF)

S6 Fig Co-staining with Golgi apparatus and ER probes in A549 cells. A549 cells were stained with a Golgi apparatus probe and ER probe, followed by treatment with 50 μM TAP-4PH for 30 min. and then observed by fluorescence microscopy. Merged image was constructed with images of TAP-4PH (cyan), Golgi apparatus probe (yellow), and ER probe (magenta). Scale bar: 20 μm.
(TIF)

S7 Fig TAP-4PH visualization of differentiated 3T3-L1 adipocytes. 3T3-L1 cells were induced to differentiate into adipocytes for 8 days. The cells were then treated with 50 μM TAP-4PH for 30 min and observed by fluorescence microscopy. Scale bar: 10 μm.
(TIF)

S8 Fig Fluorescence emission spectra of 10 μM TAP-4PH with the indicated concentrations of DNA in PBS. (TIF)
