# Supplementary figures and images for: Effective Cellular Morphology Analysis for Differentiation Processes by a Fluorescent 1,3a,6a-Triazapentalene Derivative Probe in Live Cells
Source: PLoS One. 2016 Aug 4;11(8):e0160625. doi: 10.1371/journal.pone.0160625 (PMC4973928; doi:10.1371/journal.pone.0160625)

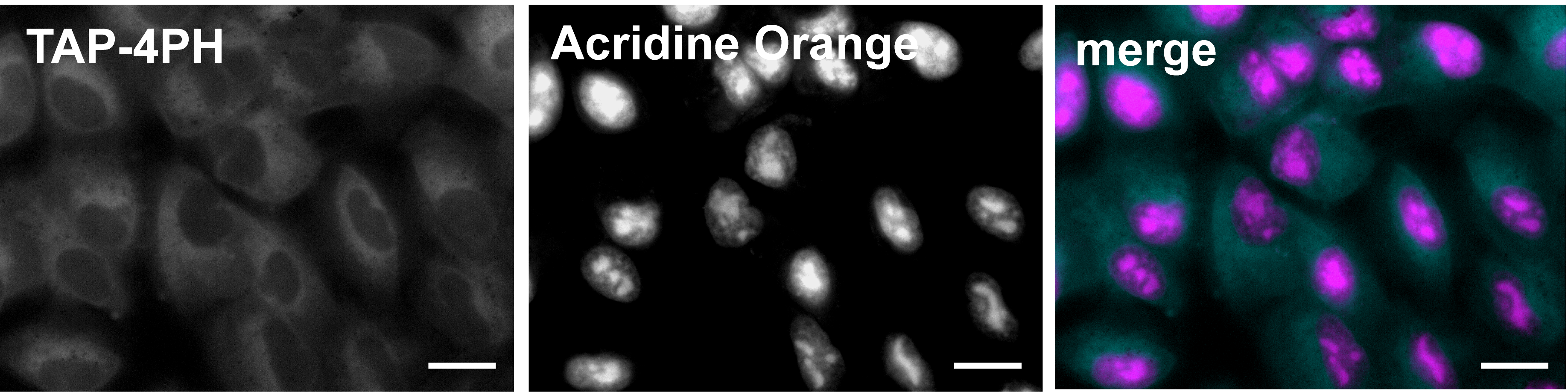

Supplement: S1 Fig — Left panel, TAP-4PH; middle panel, acridine orange; right panel, merged images of TAP-4PH (cyan) and acridine orange (magenta). Scale bar: 20 μm. (TIF) [file pone.0160625.s001.tif]

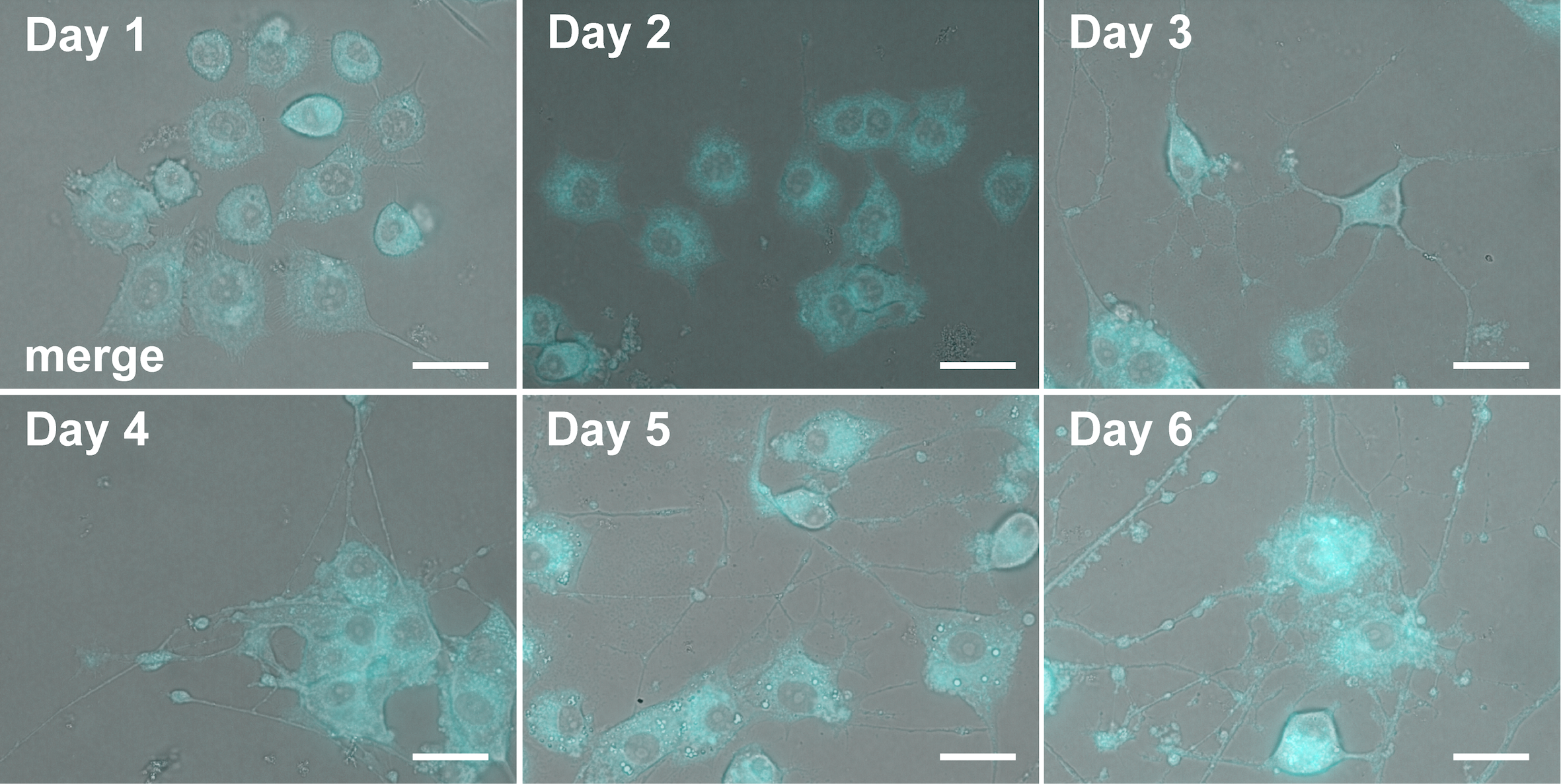

Supplement: S2 Fig — Merged bright-field and TAP-4PH fluorescent (cyan) images of PC-12 cells. (TIF) [file pone.0160625.s002.tif]

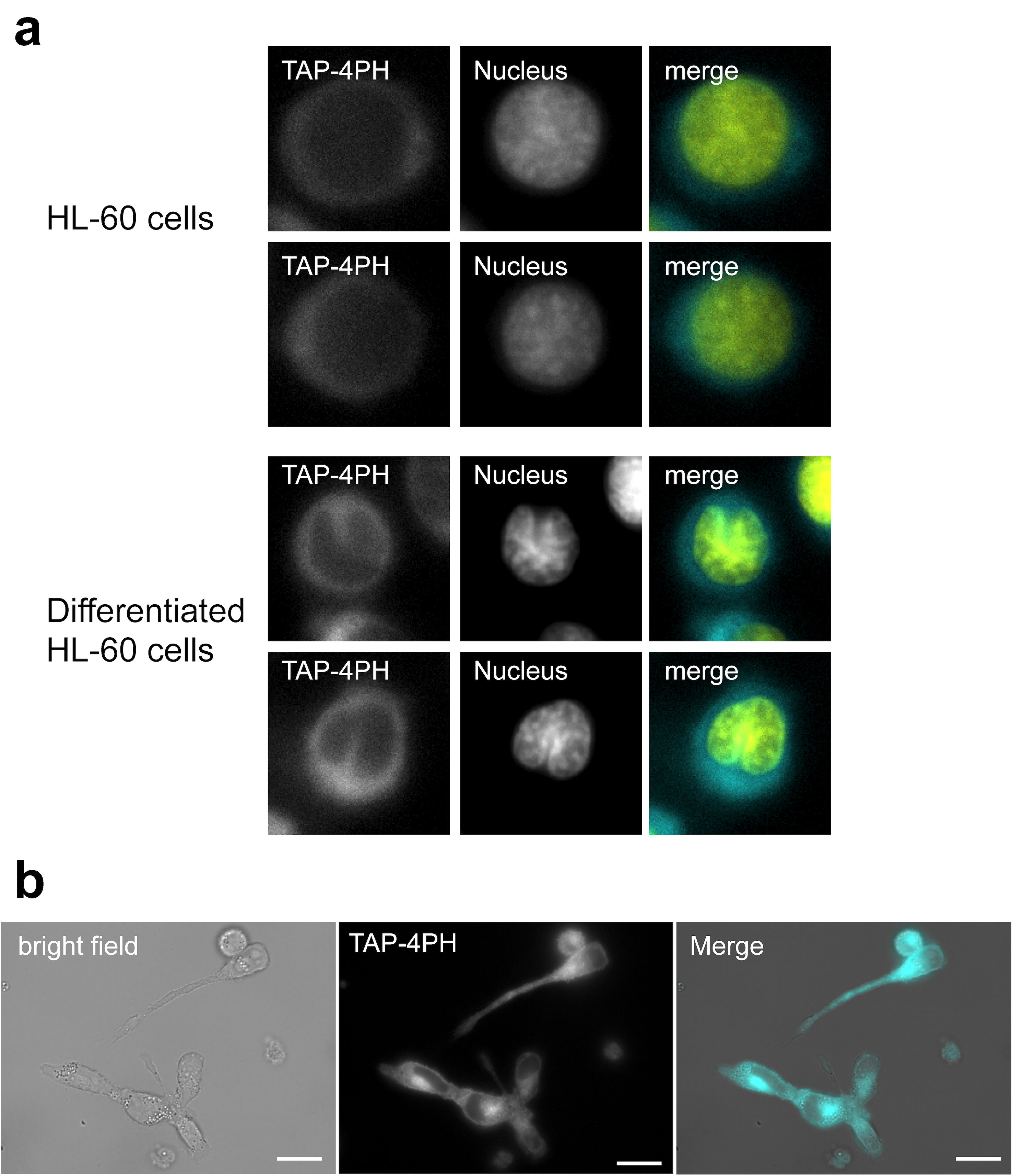

Supplement: S3 Fig — (a) HL-60 cells before and after differentiation into neutrophil-like cells by ATRA for 5 days. After differentiation, the cells were co-stained with Nuclear Green LSC1 for nucleus and TAP-4PH. Left panels, TAP-4PH; middle panels, Nuclear Green LSC1; right panels, merged images of TAP-4PH (cyan) and Nuclear Green LSC1 (yellow). (b) HL-60 cells before and after differentiation into macrophage-like cells by PMA for 48 h. After differentiation, the cells were treated with 50 μM TAP-4PH for 30 min and then observed by fluorescence microscopy. Left panel, bright field; middle panel, TAP-4PH; right panel, merged image of bright field and TAP-4PH (cyan). Scale bar: 20 μm. (TIF) [file pone.0160625.s003.tif]

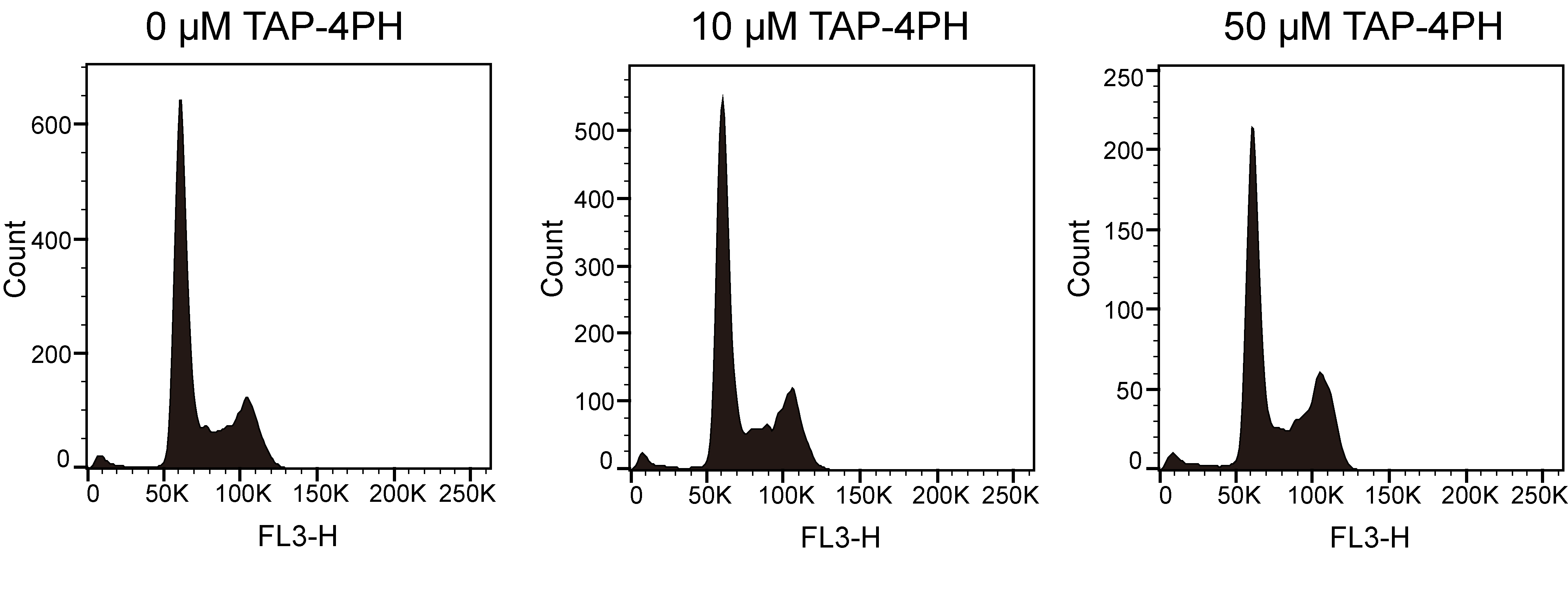

Supplement: S4 Fig — Data shown are representative of three independent experiments. (TIF) [file pone.0160625.s004.tif]

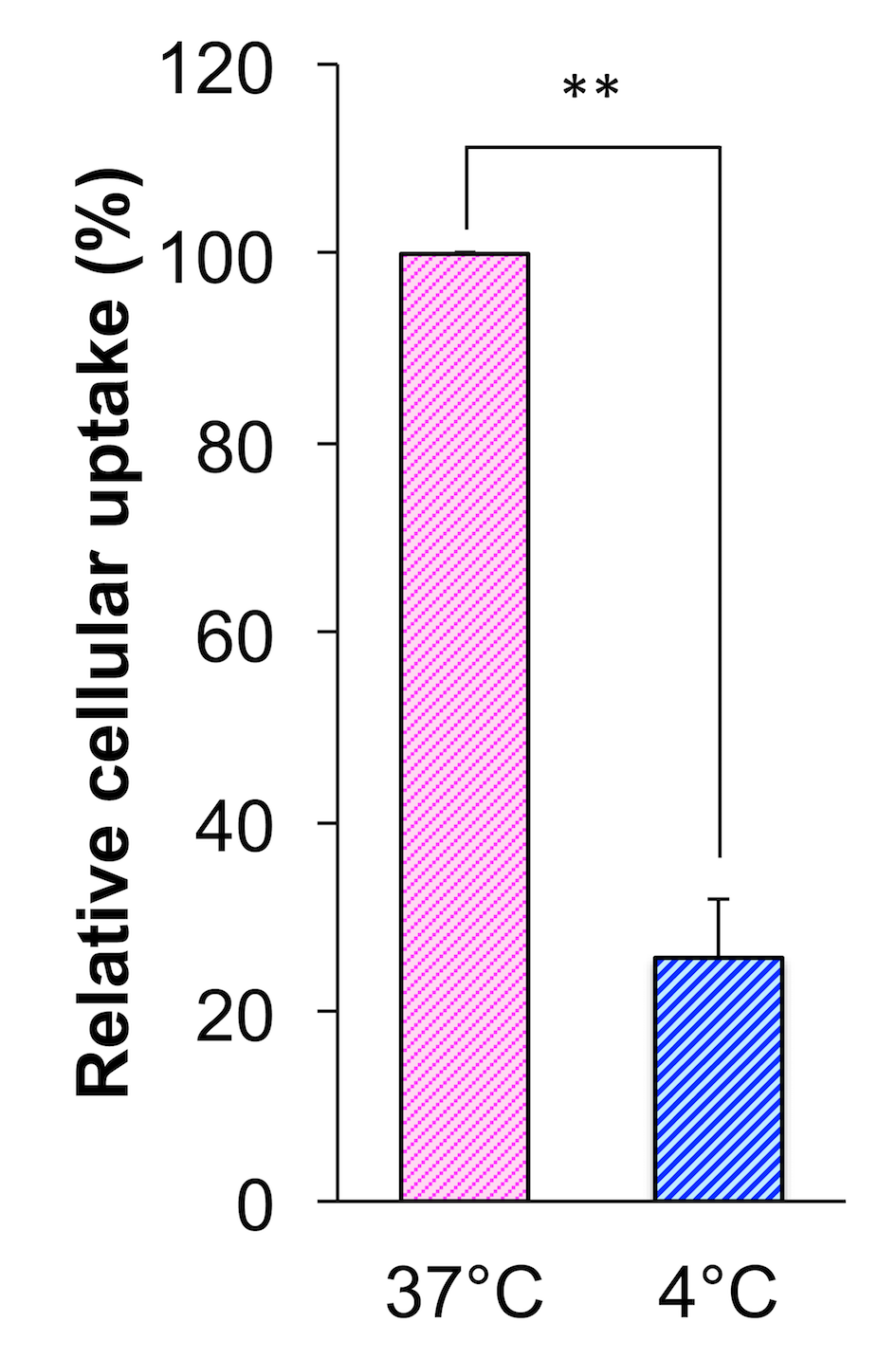

Supplement: S5 Fig — HL-60 cells were incubated with 50 μM TAP-4PH for 30 min at 37 or 4°C. Cellular uptake of TAP-4PH was measured by flow cytometric analysis. Data represent the mean ± S.D., n = 3. **p < 0.005, Student’s t-test. (TIF) [file pone.0160625.s005.tif]

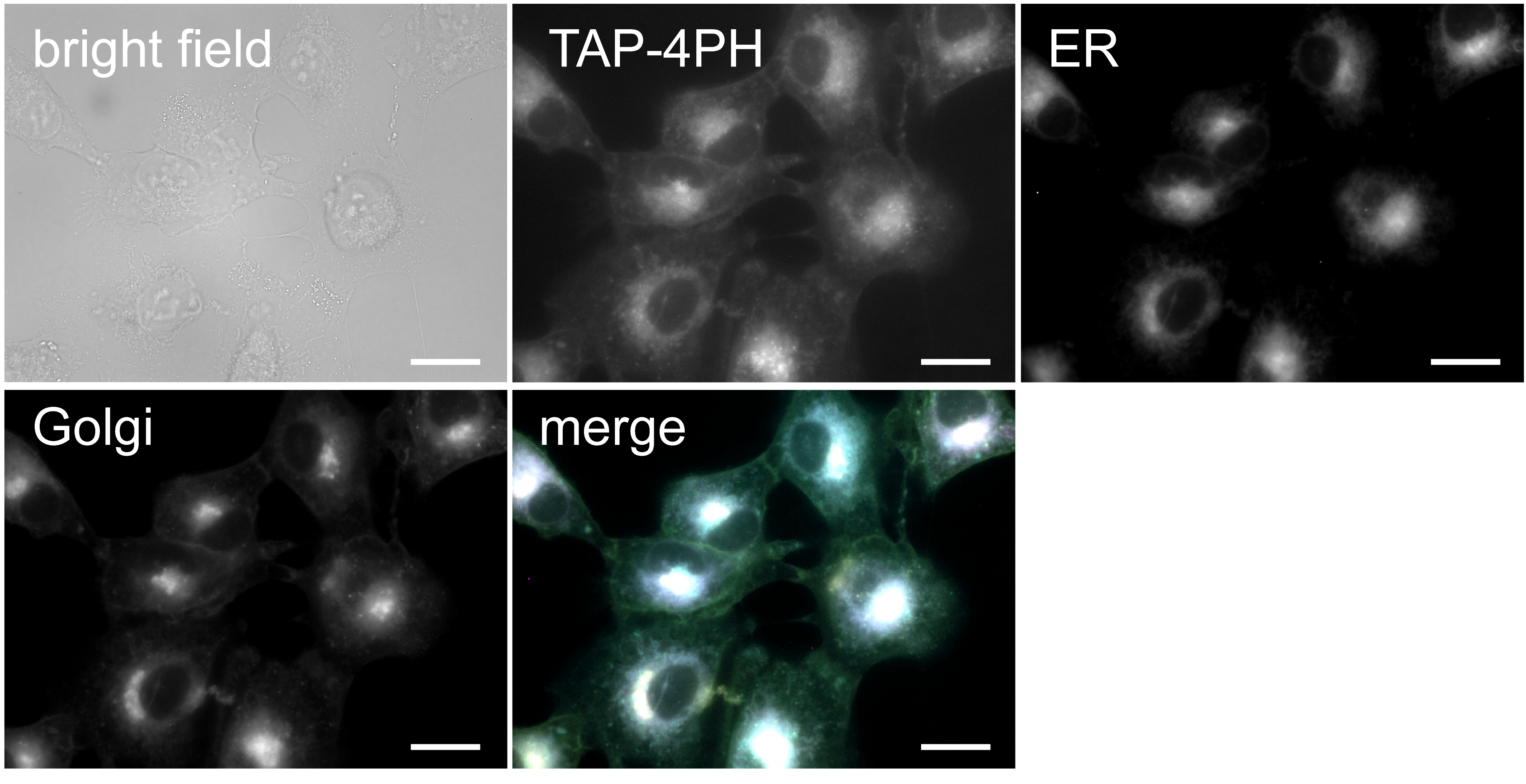

Supplement: S6 Fig — A549 cells were stained with a Golgi apparatus probe and ER probe, followed by treatment with 50 μM TAP-4PH for 30 min. and then observed by fluorescence microscopy. Merged image was constructed with images of TAP-4PH (cyan), Golgi apparatus probe (yellow), and ER probe (magenta). Scale bar: 20 μm. (TIF) [file pone.0160625.s006.tif]

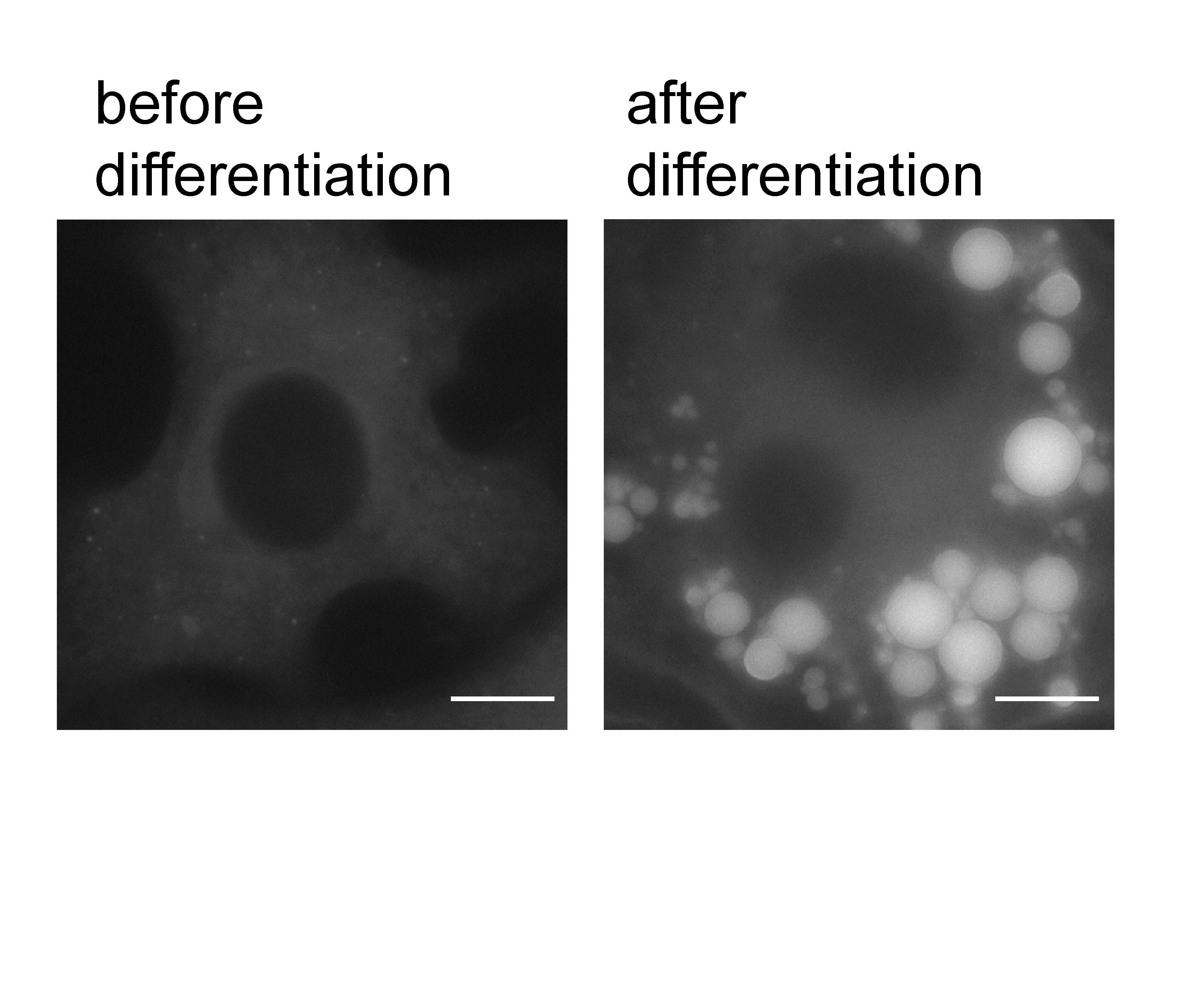

Supplement: S7 Fig — 3T3-L1 cells were induced to differentiate into adipocytes for 8 days. The cells were then treated with 50 μM TAP-4PH for 30 min and observed by fluorescence microscopy. Scale bar: 10 μm. (TIF) [file pone.0160625.s007.tif]

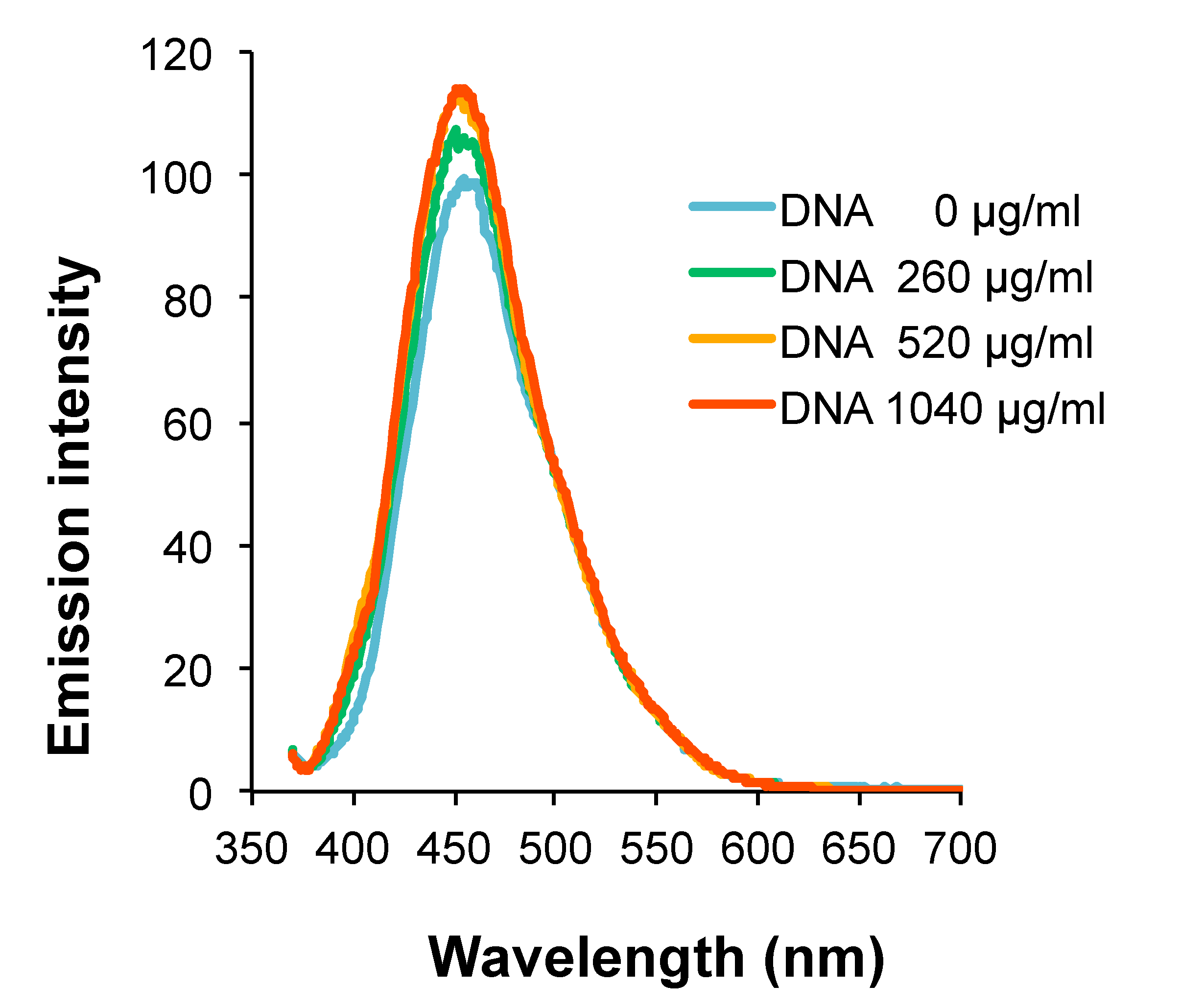

Supplement: S8 Fig — (TIF) [file pone.0160625.s008.tif]
